# Associations between physical inactivity and sedentary behaviors among adolescents in 10 cities in China

**DOI:** 10.1186/1471-2458-14-744

**Published:** 2014-07-22

**Authors:** You Chen, Zhonghui Zheng, Jinyao Yi, Shuqiao Yao

**Affiliations:** 1 Department of Orthopedic Surgery, Second Xiangya Hospital, Central South University, #139 Ren-Min Zhong Road, Changsha 410011, China; 2 Medical Psychological Institute, Second Xiangya Hospital, Central South University, #139 Ren-Min Zhong Road, Changsha 410011, China

**Keywords:** Adolescent, Physical activity, Computer, Television

## Abstract

**Background:**

Studies in western countries have revealed that excessive sedentary behavior is a major risk factor for physical inactivity in adolescents. This study was performed to investigate the association between sedentary behavior and physical inactivity in Chinese adolescents using a large-scale cross-sectional survey design.

**Methods:**

This study was part of the 2011 Chinese Youth Risk Behavior Survey. Between March and September 2011, 10,214 11–18-year-olds were recruited for survey participation in 18 schools in 10 cities in China. Demographic and socioeconomic characteristics, and the prevalences of physical inactivity and sedentary behaviors, were examined. Correlations between sedentary behavior and physical inactivity were analyzed using baseline logistic regression.

**Results:**

Among the final 9,901 students, physical inactivity (~80%) and sedentary behaviors (television viewing, 43%; computer use, 30.2%) were prevalent. More male than female students reported sedentary behaviors (television viewing > 2 h: 5.5% *vs.* 3.9%; computer use > 2 h: 7.2% *vs.* 3.5%; both *p* < 0.05), but more males were physically active than females (25.1% *vs.*14.6%; *p* < 0.05). Television viewing was associated with lower odds of no physical activity (No PA) in males [0–2 h: adjusted odds ratio (AOR) = 0.81, 95% confidence interval (CI) = 0.68–0.96; >4 h: OR = 0.34, 95% CI = 0.18–0.64], but not in females. A similar pattern between insufficient physical activity and >4 h TV viewing (AOR = 0.42, 95% CI = 0.23–0.76) and >4 h computer use (AOR = 0.49, 95% CI = 0.30–0.78) was observed in males. In females, 0–2 h daily computer use was associated with higher odds of physical inactivity (No PA: AOR = 1.42, 95% CI = 1.10–1.82; Insufficient PA: AOR = 1.58, 95% CI = 1.24–2.01), while TV viewing was not associated with No PA or Insufficient PA. The probability of physical inactivity significantly increased with grade and decreased with socioeconomic status.

**Conclusions:**

Physical inactivity and sedentary behaviors were prevalent in Chinese adolescents. Further support, including parental guidance and the provision of publicly accessible facilities, is necessary to encourage Chinese youths to engage sufficiently in physical activities.

## Background

Physical inactivity among children and adolescents has become a global health concern because it has been strongly associated with many major diseases, such as obesity, diabetes, and cardiovascular disease [1-3]. In adolescents, physical activity (PA), defined as accumulation of at least 60 min of moderate-intensity physical activity (MVPA) daily [4], is critical to attain and maintain appropriate bone strength and normal skeletal development [5]. Young adults who were physically inactive during adolescence are also more likely to be overweight than are their physically active counterparts [6]. The prevalence of physical inactivity, defined as not meeting the physical activity recommendation, is growing rapidly in adolescents [7,8], underscoring the importance of identifying and understanding associated risk factors.

One hypothesis proposed to explain the pattern of physical inactivity in adolescents holds that sedentary behaviors (e.g., computer/video game use, television viewing) compete with social and physical activities during individuals’ free time [9,10]. Adolescents are particularly likely to prefer television viewing and computer usage, commonly called "screen time", because they consider these activities to be more entertaining than physical activity [9,10]. Koezuka et al. [11] reported that television viewing was significantly associated with a higher risk of physical inactivity in Canadian youth, and the association was stronger in females than in males. And a meta-analysis produced similar conclusion [12]. However, other studies have shown no significant association between television viewing and physical inactivity [13,14]. Diverse findings regarding the relationship between computer use and PA have also been reported. In the Canadian Community Health Survey [11], male light computer users were likely to be more physically active than non-users, but their activity status did not differ from that of higher-level users. In females, physical inactivity was not significantly affected by any level of computer usage [11]. Another study conducted in Canada produced a similar conclusion that computer usage increased the probability of being physically active, but this finding applied to both males and females [13]. However, no association between computer/internet use and physical inactivity was found among adolescents in Singapore [15]. Other researchers have argued that computer use prevents youths from engaging in physical and social activities [16,17]. One potential explanation for these discrepancies is differences in socioeconomic status (SES) among cohorts, which are very likely to affect the availability of advanced technological devices. Indeed, the findings of recent studies have suggested that PA is positively associated with SES [18].

The increased prevalence of sedentary behavior and physical inactivity, as well as related health concerns, is not restricted to western countries. Rates of overweight, obesity, and physical inactivity are increasing in China, where cultural and economic patterns are distinct from those of developed countries [19]. The risks of these conditions are highest in China’s rapidly growing cities and are exacerbated by current urban development practices and policies, including support for decentralization in land use planning, neighborhood gating, and policies and practices related to motor vehicle travel, transit planning, and bicycle and pedestrian infrastructure [20]. However, few studies of the relationship between physical inactivity and sedentary behaviors have been conducted in developing countries, such as China. Exploration of the mechanisms underlying this relationship is critical for preventive purposes.

In this study, which formed part of the 2011 Chinese Youth Risk Behavior Survey, a cross-sectional survey design was used to (a) describe the prevalence of physical inactivity and sedentary behavior among Chinese urban adolescents and (b) examine potential risk factors such as socio-economic status (SES) and sedentary behaviors, associated with physical inactivity among Chinese urban adolescents.

## Methods

### Participants

A cross-sectional survey design was used to recruit high-school students from March to September 2011. Participants were recruited from high schools in 10 cities in mainland China representing three economic growth levels: high (Beijing, Shanghai, and Guangzhou), moderate (Hangzhou, Suzhou, Chengdu, and Changsha), and low (Yinchuan, Shenyang, and Changping). Schools in these cities were stratified into categories representing high, medium, and low quality of education, which was based on academic performance. Then, one to two schools in each city with a medium-level quality of education were selected, and one to three classes in each grade were selected randomly in each school. Finally, 10,214 high school students (aged 11–18 years) in 18 schools attended anonymous survey. The ethics committee of the Second Xiangya Hospital of Central South University approved the study, and all participants and their parents provided written informed consent before the survey began.

### Measures

#### Outcome: physical activity

The survey included one question from the Youth Risk Behavior Surveillance System (YRBSS) [21], developed by the Centers for Disease Control to monitor priority health-risk behaviors in youth: "During the past 7 days, on how many days were you physically active for a total of at least 60 minutes per day? (Add up all the time you spent in any kind of physical activity that increased your heart rate and made you breathe hard some of the time)." The cutoff point of 60 min was chosen because the Physical Activity Guidelines for Americans recommend that children and adolescents participate in moderate to vigorous physical activity (MVPA) for at least this long on most days of the week, preferably daily, to attain health benefits [3,22].

Estimation of PA was based on the self-reported number of days in a typical week on which respondents performed >60 min MVPA. Values were categorized as "no PA" (MVPA on 0 days), "insufficient PA" (MVPA on 1–4 days), and "PA" (MVPA on ≥5 days).

#### Primary predictors: sedentary behaviors

Two YRBSS items were used to assess sedentary behaviors: "On an average school day, how many hours do you watch TV?" and "On an average school day, how many hours do you play video or computer games or use a computer for something that is not school work? (Include activities such as Xbox, PlayStation, Nintendo DS, iPod touch, Facebook, and the Internet)." Time spent on each activity was classified on a seven-point scale ranging from none to ≥5 h. In the analysis, these categories were collapsed according to the distribution of responses, and time spent on sedentary behaviors was recoded as (i) none, (ii) > 0–2 hours, (iii) >2-4 hours, (iv) > 4 hours.

#### Covariates: potential risk factors

As potential risk factors for physical inactivity, demographic characteristics, body mass index (BMI), and SES were included as covariates to adjust for possible confounding effects. Respondents’ grade levels at the time of the survey were classified as 7th–9th (junior stage) and 10th–12th (senior stage). BMI was categorized as low/normal, overweight, and obese based on the Body Mass Index Reference for Screening Overweight and Obesity in Chinese School-age Children [23].

Objective SES indicators included family income and parents’ education levels. Monthly family incomes were coded as <1000 RMB (1), 1000–1500 RMB (2), 1500–2000 RMB (3), 2000–3000 RMB (4), 3000–4000 RMB (5), 4000–5000 RMB (6), and > RMB 5000 (7). Parents’ education levels were classified as "at least some primary school", "completed middle school", "completed high school", and "completed higher education".

Participants subjectively rated their SES in the province and the school using the Chinese version of the Scale of Subjective Status [24,25]. Responses were structured by a scale ranging from 1 to 10, with 10 representing the highest SES (most money, best education, and most respected) and 1 representing the lowest SES (least money, minimal education, and least respected). Subjective SES scores were categorized as low (1, 2), low middle (3, 4), middle (5, 6), high middle (7, 8), and high (9, 10).

### Statistical analysis

All statistical analyses were stratified by gender, given differences in behavioral patterns reported in previous studies [26,27]. Frequencies and prevalence rates are reported for categorical variables (Tables 1 and 2). Differences in demographic characteristics and prevalence rates between gender groups were analyzed by chi-squared statistic. Analyses were performed using SAS software (ver. 9.2; SAS Institute Inc., Cary, NC, USA). Statistical significance was evaluated at the 0.05 level.

**Table 1:** Descriptive characteristics of the study sample

**Variables**	**Male (*N*** **= 5057)**		**Female (*N*** **= 4844)**		**Total (*N*** **= 9901)**	
	**n**	**%**	**n**	**%**	**n**	**%**
**Grade**						
7th-9th	2587	51.2	2389	49.3	4976	50.3
10th-12th	2470	48.8	2455	50.7	4925	49.7
BMI categories*						
Low or normal	4149	82.0	4483	92.5	8623	87.2
Overweight	570	11.3	245	5.1	815	8.2
Obesity	338	6.7	116	2.4	454	4.6
**Paternal level of education**						
Complete primary school or not	1096	21.7	984	20.3	2080	21.0
Complete middle school	1566	31.0	1599	33.0	3165	32.0
Complete high school	1472	29.1	1369	28.3	2841	28.7
Complete higher education	923	18.3	892	18.4	1815	18.3
**Maternal level of education**						
Complete primary school or not	1321	26.1	1191	24.6	2512	25.4
Complete middle school	1702	33.7	1689	34.9	3391	34.2
Complete high school	1328	26.3	1259	26.0	2587	26.1
Complete higher education	706	14.0	705	14.6	1411	14.3
**Household income per month (￥)**						
<1000	233	4.6	211	4.4	444	4.5
1000-1500	498	9.8	518	10.7	1016	10.3
1500-2000	530	10.5	587	12.1	1117	11.3
2000-3000	893	17.7	862	17.8	1755	17.7
3000-4000	1001	19.8	928	19.2	1929	19.5
4000-5000	723	14.3	760	15.7	1483	15.0
>5000	1179	23.3	978	20.2	2157	21.8
**Subject economic status (in province)**						
Low	74	1.5	59	1.2	133	1.3
Low middle	554	11.0	516	10.7	1070	10.8
Middle	2447	48.4	2466	50.9	4913	49.6
High middle	1592	31.5	1433	29.6	3025	30.6
High	390	7.7	370	7.6	760	7.7
Subject economic status (in school)*						
Low	76	1.5	35	0.7	111	1.1
Low middle	361	7.1	259	5.3	620	6.3
Middle	2176	43.0	2175	44.9	4351	43.9
High middle	1996	39.5	1939	40.0	3935	39.7
High	448	8.9	436	9.0	884	3.9

*Significant difference between male and female, p < 0.05.

**Table 2:** Prevalence of physical inactivity and sedentary behaviors

**Variables**	**Male (*N*** **= 5057)**		**Female (*N*** **= 4844)**		**Total (*N*** **= 9901)**	
	**n**	**%**	**n**	**%**	**n**	**%**
Physical activity/inactivity time*						
None	1605	31.7	2345	48.4	3950	39.9
Insufficient	2185	43.2	1794	37.0	3979	40.2
Active	1267	25.1	705	14.6	1972	19.9
Television viewing*						
None	2634	52.1	3013	62.2	5647	57.0
>0-2 hours	2142	42.4	1643	33.9	3785	38.2
>2-4 hours	204	4.0	145	3.0	349	3.5
>4 hours	77	1.5	43	0.9	120	1.2
Computer use*						
None	3339	66.0	3572	73.7	6911	69.8
>0-2 hours	1355	26.8	1100	22.7	2455	24.8
>2-4 hours	237	4.7	127	2.6	364	3.7
>4 hours	126	2.5	45	0.9	171	1.7

*Significant difference between male and female, p < 0.05.

Baseline logistic regression was performed to estimate the association between physical inactivity and sedentary behaviors, with "physically active" serving as the reference level for the outcome. BMI, parents’ education levels, monthly income, and subjective SES were treated as ordinal variables. First, each sedentary activity was separately added to the model, followed by adjustment for covariates. Then, the model was examined with the inclusion of both sedentary behaviors.

## Results

### Descriptive characteristics

List-wise deletion due to missing responses resulted in the exclusion of 313 students. Data from 9,901 students in the 7th–12th grades [5,057 (51.8%) males, 4,844 (48.2%) females] were included in the final analysis. The final 9,901 students ranged in age from 11 to 18 years (15.06 ± 1.81), and the age difference between male (15.09 ± 1.80) and female (15.03 ± 1.82) was not significant, *p* > 0.05. Sample characteristics are summarized in Table 1. No marked difference in descriptive characteristics was observed between the excluded group and the entire study sample (all *p* > 0.05), which supported the representativeness of the study sample (data not shown).

Higher prevalences of overweight and obesity were observed in male than in female students (11.3% *vs*. 5.1% and 6.7% *vs*. 2.4%, respectively; both *p* < 0.05). Most covariates associated with SES were similar between genders, except more male than female students rated themselves having low SES in school (Table 1).

### The prevalence of PA

The prevalence of achieving recommended PA levels was 19.9% for all participants; more than one-third (39.9%) of participants performed <1 h PA during the past 7 days. There was a significant difference in the physical activity/inactivity variables between the males and females (*p* < 0.05) with 25.1% of males being categorized as active compared to 14.6% of females (Table 2).

### The prevalence of sedentary behavior

Approximately 43% of adolescents reported watching television and 30.2% reported computer usage (Table 2). There were significant differences in the television viewing/computer use variables between the males and females (*ps* < 0.05); 5.5% of males watched television for >2 h every day, whereas only 3.9% of females reported this behavior; 7.2% of males reported that they used the computer for >2 h every day, compared with 3.5% of females (Table 2).

### Factors correlated with physical inactivity

Baseline univariate logistic models investigating the associations between physical inactivity and the independent variables (Tables 3 and 4) revealed that high-school students (10th–12th grades) were more likely than middle-school students (7th–9th grades) to engage in no PA. Generally, students with higher SES, which included better-educated parents, higher monthly family incomes, and higher subjective SES in the province and the school, were less likely to do no physical activity. BMI was not significantly associated with PA in males or females. In male students, 0–2 h/day television viewing was associated with decreased odds of doing no PA (0 hours MVPA/week) and an increased odds of being insufficiently active. In addition, >4 h television viewing was associated with decreased odds of doing no physical activity and being insufficiently active in male students. Television viewing was not significantly associated with participating in no physical activity or insufficient physical activity in female students. More than 4 h daily computer use was associated with lower odds of no PA and insufficient physical activity in males, but not in females. Conversely, less (0–2 h daily) computer usage was associated with higher odds of physical inactivity in females, but not in males.

**Table 3:** Odds ratios (OR) and 95% confidence intervals (95% CI) of physical inactivity by sedentary behaviors for males (N = 5057)

	**No PA**^**1**^				**Insufficient PA**^**2**^			
	**Univariate**		**Multivariate**		**Univariate**		**Multivariate**	
	**OR**	**95% CI**	**OR**	**95% CI**	**OR**	**95% CI**	**OR**	**95% CI**
**Grade**								
7th-9th	1.00		1.00		1.00		1.00	
10th-12th	5.72*	(4.86, 6.72)	5.48*	(4.64, 6.47)	2.27*	(1.96, 2.64)	2.26*	(1.94, 2.64)
**BMI**								
Low/normal	1.00		1.00		1.00	-	1.00	
Overweight	0.88	(0.70, 1.11)	0.95	(0.74, 1.22)	0.91	(0.73, 1.13)	0.93	(0.75, 1.16)
Obese	1.00	(0.75, 1.34)	1.21	(0.88, 1.65)	0.91	(0.69, 1.20)	0.97	(0.73, 1.29)
**Paternal level of education**								
Some primary school	1.00		1.00		1.00	-	1.00	
Completed middle school	0.95	(0.77, 1.17)	0.99	(0.76, 1.29)	0.83	(0.68, 1.02)	0.92	(0.72, 1.19)
Completed high school	0.70	(0.57, 0.87)	0.77	(0.58, 1.02)	0.84	(0.69, 1.02)	0.97	(0.75, 1.27)
Completed higher education	0.58*	(0.46, 0.74)	0.86	(0.60, 1.23)	0.75	(0.61, 0.93)	0.96	(0.70, 1.32)
**Maternal level of education**								
Some primary school	1.00		1.00		1.00	-	1.00	
Completed middle school	0.93	(0.77, 1.13)	1.05	(0.81, 1.35)	0.83	(0.69, 1.0)	0.89	(0.70, 1.13)
Completed high school	0.73	(0.59, 0.90)	1.0	(0.75, 1.32)	0.77	(0.63, 0.93)	0.84	(0.65, 1.09)
Completed higher education	0.58*	(0.45, 0.74)	1.08	(0.75, 1.55)	0.75	(0.60, 0.94)	1.02	(0.74, 1.41)
**Monthly family income**								
<1000 RMB	1.00		1.00		1.00	-	1.00	
1000–1500 RMB	1.00	(0.64, 1.55)	1.13	(0.70, 1.82)	0.97	(0.62, 1.51)	0.97	(0.61, 1.55)
1500–2000 RMB	0.52	(0.34, 0.80)	0.60	(0.37, 0.96)	0.84	(0.55, 1.29)	0.86	(0.55, 1.36)
2000–3000 RMB	0.65	(0.43, 0.97)	0.81	(0.51, 1.27)	0.95	(0.64, 1.43)	0.98	(0.63, 1.52)
3000–4000 RMB	0.52	(0.35, 0.78)	0.71	(0.45, 1.10)	0.76	(0.51, 1.13)	0.81	(0.53, 1.25)
4000–5000 RMB	0.51	(0.34, 0.78)	0.78	(0.49, 1.24)	0.81	(0.53, 1.21)	0.91	(0.58, 1.42)
>RMB 5000	0.32*	(0.21, 0.47)	0.49*	(0.31, 0.77)	0.55*	(0.37, 0.81)	0.62	(0.40, 0.95)
**Subjective SES in province**								
Low	1.00		1.00		1.00	-	1.00	-
Low middle	1.11	(0.56, 2.19)	1.35	(0.63, 2.90)	0.79	(0.41, 1.53)	0.78	(0.38, 1.60)
Middle	0.78	(0.41, 1.53)	1.29	(0.61, 2.74)	0.83	(0.44, 1.56)	0.94	(0.47, 1.92)
High middle	0.48*	(0.25, 0.92)	1.28	(0.59, 2.74)	0.67	(0.35, 1.26)	1.04	(0.51, 2.14)
High	0.37*	(0.18, 0.73)	1.44	(0.64, 3.23)	0.49*	(0.25, 0.96)	1.01	(0.48, 2.15)
**Subjective SES in school**								
Low	1.00		1.00		1.00	-	1.00	-
Low middle	0.79	(0.40, 1.55)	0.71	(0.34, 1.50)	0.86	(0.44, 1.68)	0.84	(0.41, 1.70)
Middle	0.89	(0.47, 1.69)	0.74	(0.37, 1.49)	1.04	(0.56, 1.94)	0.91	(0.46, 1.77)
High middle	0.51	(0.27, 0.97)	0.44*	(0.22, 0.89)	0.71	(0.38, 1.32)	0.61	(0.31, 1.19)
High	0.41*	(0.21, 0.79)	0.45*	(0.22, 0.95)	0.48*	(0.25, 0.93)	0.48*	(0.24, 0.96)
**Television viewing**								
None	1.00		1.00		1.00		1.00	
>0-2 hours	0.81*	(0.69, 0.95)	0.81*	(0.68, 0.96)	1.21*	(1.05, 1.39)	1.21*	(1.04, 1.41)
>2-4 hours	0.70	(0.49, 1.00)	0.64	(0.43, 0.96)	0.73	(0.51, 1.03)	0.75	(0.52, 1.09)
>4 hours	0.40*	(0.23, 0.70)	0.34*	(0.18, 0.64)	0.38*	(0.22, 0.66)	0.42*	(0.23, 0.76)
**Computer use**								
None	1.00		1.00		1.00		1.00	
>0-2 hours	1.04	(0.88, 1.24)	1.06	(0.88, 1.28)	1.08	(0.92, 1.27)	1.00	(0.84, 1.18)
>2-4 hours	0.96	(0.69, 1.35)	1.04	(0.71, 1.51)	0.81	(0.58, 1.12)	0.82	(0.58, 1.16)
>4 hours	0.56*	(0.36, 0.86)	0.64	(0.39, 1.05)	0.41*	(0.27, 0.63)	0.49*	(0.30, 0.78)

Covariate: Grade, BMI, Paternal level of education, Maternal level of education, Monthly family income, Subjective SES in province, Subjective SES in school.

^1^MVPA on 0 days: spent 0 day out of the past 7 days with at least 60 minutes of moderate-to-vigorous physical activity (MVPA).

^2^MVPA on 1–4 days: spent less than 5 days out of the past 7 days with at least 60 minutes of MVPA.

*p < 0.05.

**Table 4:** Odds ratios (OR) and 95% confidence intervals (95% CI) of physical inactivity by sedentary behaviors for females (N = 4844)

	**No PA**^**1**^				**Insufficient PA**^**2**^			
	**Univariate**		**Multivariate**		**Univariate**		**Multivariate**	
	**OR**	**95% CI**	**OR**	**95% CI**	**OR**	**95% CI**	**OR**	**95% CI**
**Grade**								
7th-9th	1.00		1.00		1.00		1.00	
10th-12th	16.05*	(12.82, 20.08)	15.06*	(11.98, 18.94)	2.73*	(2.17, 3.42)	2.65*	(2.11, 3.34)
**BMI**								
Low/normal	1.00		1.00		1.00	-	1.00	
Overweight	1.32	(0.87, 1.99)	1.00	(0.63, 1.57)	1.23	(0.80, 1.89)	1.11	(0.71, 1.70)
Obese	0.77	(0.45, 1.31)	1.28	(0.71, 2.30)	1.02	(0.60, 1.75)	1.12	(0.65, 1.95)
**Paternal level of education**								
Some primary school	1.00		1.00		1.00	-	1.00	
Completed middle school	1.05	(0.82, 1.34)	1.09	(0.79, 1.50)	0.95	(0.73, 1.22)	0.92	(0.67, 1.25)
Completed high school	0.91	(0.71, 1.66)	1.35	(0.95, 1.92)	0.97	(0.75, 1.26)	1.05	(0.75, 1.47)
Completed higher education	0.59*	(0.45, 0.77)	1.37	(0.91, 2.05)	0.88	(0.67, 1.15)	1.06	(0.72. 1.56)
**Maternal level of education**								
Some primary school	1.00		1.00		1.00	-	1.00	
Completed middle school	1.03	(0.82, 1.29)	1.01	(0.75, 1.37)	1.02	(0.80, 1.30)	1.08	(0.80, 1.45)
Completed high school	0.73	(0.57, 0.92)	0.88	(0.62, 1.23)	0.91	(0.71, 1.64)	1.05	(0.75, 1.45)
Completed higher education	0.47*	(0.36, 0.63)	0.89	(0.59, 1.34)	0.99	(0.75, 1.30)	1.41	(0.96, 2.07)
**Monthly family income**								
<1000 RMB	1.00		1.00		1.00	-	1.00	
1000–1500 RMB	1.00	(0.57, 1.75)	1.07	(0.63, 2.16)	0.84	(0.47, 1.51)	0.85	(0.46, 1.55)
1500–2000 RMB	0.84	(0.49, 1.46)	1.04	(0.57, 1.92)	0.95	(0.54, 1.68)	0.98	(0.54, 1.77)
2000–3000 RMB	0.62	(0.37, 1.03)	0.78	(0.44, 1.41)	0.71	(0.42, 1.22)	0.72	(0.41, 1.26)
3000–4000 RMB	0.67	(0.40, 1.11)	0.92	(0.51, 1.65)	0.76	(0.45, 1.30 )	0.75	(0.43, 1.34)
4000–5000 RMB	0.56	(0.34, 0.95)	0.82	(0.45, 1.50)	0.64	(0.37, 1.09)	0.63	(0.35, 1.13)
>RMB 5000	0.32*	(0.19, 0.53)	0.48*	(0.27, 0.87)	0.43*	(0.26, 0.73)	0.44*	(0.25, 0.78)
**Subjective SES in province**								
Low	1.00		1.00		1.00	-	1.00	
Low middle	0.92	(0.34, 2.45)	1.18	(0.40, 3.52)	1.10	(0.38, 3.16)	1.24	(0.41, 3.77)
Middle	0.56	(0.22, 1.43)	1.01	(0.35, 2.95)	0.93	(0.34, 2.56)	1.21	(0.41, 3.60)
High middle	0.30*	(0.12, 0.76)	0.88	(0.30, 2.59)	0.70	(0.25, 1.92)	1.14	(0.38, 3.43)
High	0.19*	(0.07, 0.50)	0.92	(0.30, 2.79)	0.48	(0.17, 1.37)	0.94	(0.31, 2.90)
**Subjective SES in school**								
Low	1.00		1.00		1.00	-	1.00	
Low middle	1.27	(0.47, 3.41)	0.94	(0.32, 2.80)	1.92	(0.63, 5.81)	1.86	(0.59, 5.86)
Middle	1.26	(0.50, 3.17)	0.68	(0.25, 1.90)	1.94	(0.68, 5.50)	1.79	(0.60, 5.33)
High middle	0.91	(0.36, 2.28)	0.63	(0.23, 1.76)	1.61	(0.57, 4.56)	1.68	(0.57, 5.00)
High	0.47*	(0.18, 1.20)	0.49	(0.17, 1, 40)	1.27	(0.44, 3.66)	1.54	(0.51, 4.66)
**Television viewing**								
None	1.00		1.00		1.00		1.00	
>0-2 hours	0.91	(0.76, 1.09)	0.83	(0.67, 1.03)	1.18	(0.98, 1.42)	1.02	(0.84, 1.25)
>2-4 hours	0.67	(0.42, 1.06)	0.68	(0.40, 1.17)	0.77	(0.48, 1.25)	0.73	(0.44, 1.21)
>4 hours	1.30	(0.53, 3.16)	1.21	(0.44, 3.30)	0.69	(0.25, 1.90)	0.63	(0.22, 1.81)
**Computer use**								
None	1.00		1.00		1.00		1.00	
>0-2 hours	1.35*	(1.09, 1.68)	1.42*	(1.10, 1.82)	1.57*	(1.26, 1.96)	1.58*	(1.24, 2.01)
>2-4 hours	0.95	(0.58, 1.55)	0.96	(0.54, 1.71)	0.78	(0.46, 1.33)	0.84	(0.47, 1.47)
>4 hours	2.33	(0.81, 6.64)	2.66	(0.85, 8.37)	1.29	(0.41, 4.02)	1.53	(0.48, 4.88)

Covariate: Grade, BMI, Paternal level of education, Maternal level of education, Monthly family income, Subjective SES in province, Subjective SES in school.

^1^MVPA on 0 days: spent 0 day out of the past 7 days with at least 60 minutes of moderate-to-vigorous physical activity (MVPA).

^2^MVPA on 1–4 days: spent less than 5 days out of the past 7 days with at least 60 minutes of MVPA.

*p < 0.05.

After adjustment for covariates, the multivariate model including both sedentary behaviors showed that grade remained associated with higher odds of physical inactivity in males and females (Tables 3 and 4). Higher monthly family income continued to be associated with lower odds of doing no PA in males and females, and insufficient PA in females only. Students with higher subjective SES in school were less likely to perform no PA. Higher subjective SES in school also protected male students from being insufficient physically active (Tables 3 and 4).

Male students who watched television for 0–2 h or >4 h daily were less likely to engage in no PA compared to those with no television viewing (0 hours). In males, watching television for >4 h daily had the same effect on no PA and insufficient PA, but 0–2 h television viewing was associated with a greater probability of insufficient PA. Television viewing was not significantly associated with participating in no physical activity or insufficient physical activity in female students (Tables 3 and 4).

Light (0–2 h) computer use was associated with higher odds of no PA or insufficient PA in females, whereas >4 h daily computer use was associated with lower odds of insufficient physical activity in male students. Univariate models including single sedentary behaviors revealed patterns similar to those of the multivariate model (data not shown).

## Discussion

In this sample of Chinese youths, the overall prevalence of physical inactivity (~80%) was high. This finding was consistent with those from the United States [28] and South Africa [29]. More than 40% of participants reported television viewing and 30% reported computer use, indicating that screen time has continued to increase among Chinese adolescents since 2006 [30].

In agreement with previous research in Canada [10], this study found that patterns of PA and sedentary behaviors differed between males and females. More male students reported sedentary behaviors, including television viewing and computer use, as well as more physical insufficient activity, and being more physically active than female students, but television viewing was no significantly associated with physical inactivity in females.

The association between screen time and PA differed between males and females. Male students who watched television were less likely to engage in no PA than males who did not watch television, but television viewing was not significantly associated with physical inactivity in females. A similar pattern was observed in male students who reported >4 h daily computer use. Female students were more sensitive to computer than to television use, as 0–2 h daily computer use increased the risk of doing no physical activity or being insufficiently active. This difference might be caused by the different purposes of sedentary behaviors.

The results of a recent study suggested that sedentary behaviors for "productive" purposes were positively associated with PA, whereas television viewing or computer use alone was not [13]. For example, males may prefer to watch sports on television or online, which could motivate them to perform PA, whereas females may prefer other types of program. Thus, further investigation of the effects of different types of sedentary pursuit on PA among Chinese youths is needed.

The findings of a previous study [10] also suggested that computer use competed with television viewing, but this phenomenon was not observed in the present study. Models including single sedentary behaviors revealed patterns similar to those of models including both behaviors, indicating that the two behaviors were independently associated with PA. Some researchers have also hypothesized that time management skills interact with sedentary behavior to influence PA in adolescents [13], but evidence obtained in the present study was insufficient to examine this issue.

High-school students were more likely than middle-school students to be physically inactive, which might be due to two major reasons. Firstly, academic pressure to prepare for the national college entrance examination may be a significant driving force in sedentary behavior. Secondly, age is a contributor, as previous studies showed that there existed an age related decline in physical activity in students [21,31]. Consistent with the findings of previous studies [19], SES was positively associated with PA level in our participants. Adolescents with higher monthly family incomes and subjective SES in school were less likely to perform no PA or to be physically inactive. Financial limitations may impact students’ access to many types of PA, such as sports requiring special equipment and motion-controlled video games. Therefore, public playgrounds are necessary to protect adolescents with lower SES from physical inactivity. Meanwhile, in this study, adolescents with better-educated parents were less likely to be physically inactive, which was consistent with previous results and suggested the parental guidance be necessary to ensure proper engagement in these behaviors [32].

This study was limited by the cross-sectional design, which prevented the identification of any causal relationship between sedentary behavior and PA. Thus, future longitudinal studies and further investigations of the association between sedentary behavior purposes and physical inactivity are needed. Meanwhile, social desirability bias might affect the subjective measures of SES, which should be paid attention to in future study.

## Conclusions

Physical inactivity and sedentary behaviors were prevalent in Chinese adolescents. Our study revealed complex relationships between sedentary behaviors and physical inactivity, highlighting the need for targeted interventions. The association between sedentary behavior and physical inactivity differed between males and females, possibly due to differences in the purposes of behaviors. Parental guidance may be necessary to ensure proper engagement in these behaviors. Public facilities are also necessary to encourage students with low SES to engage in physical activities.

## Abbreviations

BMI: Body mass index; CI: Confidence interval; MVPA: Moderate to vigorous physical activity; OR: Odds ratio; PA: Physical activity; SES: Socioeconomic status; YRBSS: Youth Risk Behavior Surveillance System.

## Competing interests

The authors declare that they have no competing of interests.

## Authors’ contributions

SY and JYwas involved in the design of the study. YC and ZZ were responsible for data collection and analyses. YC wrote the manuscript. All authors have read and approved the final version of the manuscript.

## Pre-publication history

The pre-publication history for this paper can be accessed here:

http://www.biomedcentral.com/1471-2458/14/744/prepub
